# A privacy-preserving scheme with multi-level regulation compliance for blockchain

**DOI:** 10.1038/s41598-023-50209-x

**Published:** 2024-01-03

**Authors:** Wangjing Jia, Tao Xie, Baolai Wang

**Affiliations:** 1 National University of Defense Technology, Changsha, Hunan China; 2 Shanxi Police College, Shanxi Taiyuan, China

**Keywords:** Computational science, Computer science, Information technology

## Abstract

With the increasing presence of blockchain-based distributed applications in various aspects of daily life, there has been a growing focus on the privacy protection of blockchain ledgers and the corresponding regulatory technologies. However, current mainstream solutions primarily concentrate on the verifiable encryption of blockchain transaction addresses and contents, neglecting the regulatory requirements for private transactions. Moreover, the few monitorable solutions suffer from issues such as excessive centralization and a single-minded approach to regulatory content. To address these deficiencies, this paper proposes a blockchain privacy-preserving scheme that supports multi-level regulation through the utilization of zero-knowledge proofs (zk-SNARKs) and attribute-based encryption (ABE). Firstly, by leveraging zk-SNARKs, this scheme achieves blockchain privacy-preserving within an account model, enabling the concealment of user transaction addresses and values. Secondly, by employing attribute-based encryption, a multi-level regulatory model is developed alongside the privacy protection measures, allowing for selective disclosure of transaction content. Finally, we analyze the security of the proposed scheme and compare it with other schemes, discussing its advantages in terms of privacy, security, and regulatory capabilities, we also provide a preliminary evaluation of the scheme's efficiency through experiments. In conclusion, the scheme demonstrates strong privacy by relying on mathematical proofs through zk-SNARKs to ensure security while comprehensively safeguarding content. It also achieves multi-level regulation on the foundation of privacy protection, with comprehensive regulatory coverage and decentralized regulatory authority.

## Introduction

Currently, blockchain technology is widely employed in the financial sector due to its decentralized nature, tamper-proof properties, and anonymity. However, to achieve a global synchronization, blockchain ledgers require transaction details (such as addresses, values, etc.) to be made public on the chain. This enables other users to verify the correctness of transactions and record them. While it enhances the security of the blockchain system, the public nature of ledger information compromises the privacy of users. Transaction details are accessible to anyone, allowing analysis that can potentially reveal the physical address area of the parties involved and even their real identities. This privacy breach significantly restricts the application scenarios of blockchain technology. In reality, the disclosure of transaction details is primarily aimed at ensuring the validity of transactions, but it is not an absolute necessity. As long as transaction verification can be achieved in an encrypted state, privacy protection can be preserved. Currently, various privacy protection schemes have been proposed and implemented, with the majority focusing on severing the link between the addresses of transaction parties or concealing specific transaction values. However, privacy protection can also facilitate illicit activities by malicious users, making it a challenge to trace some illegal transactions. Consequently, addressing regulatory issues has become a pressing matter for enterprises, governments, and military departments. In the future development of blockchain, striking a balance between privacy protection and regulatory compliance is of utmost importance. The regulation mechanism should address illegal data within the blockchain network through prevention, detection, tracking, and accountability measures while safeguarding the privacy information of legitimate users. As a result, finding a middle ground between privacy protection and regulation, establishing a controllable regulatory system that safeguards the privacy of honest users and tracks the information of illegal users, will emerge as one of the primary directions for future blockchain advancements.

### Challenges

Currently, most privacy protection schemes suffer from insufficient privacy and do not support account-based blockchain systems. It is difficult for some schemes to provide regulatory functions to relevant organizations while protecting user privacy. The current privacy protection schemes that can be regulated can only provide basic auditing of amount ranges and tracking of transaction addresses. The regulatory content is not comprehensive enough. All types of privacy protection schemes need to balance the relationship between privacy and regulators. Currently, most schemes are based on a single trusted third party as a regulator, resulting in excessive concentration of regulatory power.

### Contribution

To address the aforementioned issues, we propose a blockchain privacy protection scheme based on BlockMaze that supports multi-level regulation. This scheme offers the following features:

1. The use of the zk-SNARKs algorithm enables a privacy protection scheme based on an account model. This ensures the confidentiality of both account balance and transaction value, while also severing the mapping relationship between transaction parties. As a result, anonymous transactions can be achieved.
2. Building upon this privacy scheme, a multi-level regulatory structure is designed. It incorporates various roles with distinct identity attributes such as monitors, primary regulators, senior regulators, transaction parties, and miners. Each level of regulator is responsible for tracking different transaction information and has the option for real-name authentication. By distributing regulatory tasks among different entities, the harm of information leakage from a single node is mitigated, and regulatory efficiency is enhanced.
3. ABE encryption is utilized to assign keys with specific attributes to each level of regulator. Users attach transaction privacy information encrypted with ABE public keys of corresponding attributes to the transaction. This approach enables selective disclosure of transaction information and reduces the centralization prevalent in current regulatory measures.

By implementing this blockchain privacy protection scheme, we can address the aforementioned challenges while striking a balance between privacy and regulatory requirements.

The rest of this paper is organized as follows. In "Related work", we present the related work. Then, preliminaries are provided in "Preliminaries", the privacy model and Multi-level Regulatory Model are formulated in "Our scheme". In "The protocol description", the detailed construction of our protocol are described. Security analysis is given in "Security analysis" and performance evaluation is presented in "Performance analysis". Finally, we conclude the paper in "Conclusions".

## Related work

In recent years, numerous research findings have been published on privacy protection in blockchain. In 2014, Bonneau et al. introduced the Mixcoin protocol [^1^], which ensured transaction address privacy and incorporated an audit mechanism to govern third parties. Subsequently, Maxwell proposed the Coinjoin protocol ^2^ that achieved decentralized coin mixing without relying on trusted third parties, but it required participating users to negotiate and execute the mixing process themselves. To comprehensively safeguard transaction privacy, SASSON et al. proposed the ZeroCash scheme ^3^, which employed zero-knowledge proof technology to protect the addresses and transaction values of both transaction initiators and receivers. However, this scheme relied on trusted third parties for parameter initialization and suffered from low efficiency. Monero ^4^ utilized ring signature technology ^5^ to protect data privacy and employed stealth addresses ^6,7^ to hide the associativity problem between input and output addresses. Nonetheless, ring signatures had security vulnerabilities and necessitated multiple off-chain interactions to complete transactions ^8,9^. The MimbleWimble protocol ^10^, proposed by Tom Elvis Jedusor, combined mixing, encryption commitment, range proof ^11^, and Dandelion ^12^ technologies to ensure the privacy of blockchain transactions. However, it required multiple user interactions to complete private transactions, and its security was subject to debate. Subsequently, projects offering privacy protection for smart contracts on public chains began gaining prominence in various scenarios. The Hawk protocol ^13^, proposed by Kosba et al., implemented smart contract privacy protection based on zk-SNARKs. The Ekiden protocol ^14^, studied by Oasis Labs, implemented privacy computing based on TEE. The Zether protocol ^15^, introduced by Bünz et al., protected the input and output values of smart contracts. The BlockMaze ^16^ established a blockchain privacy protection solution based on zk-SNARKs for an account-based model, which was more compatible with smart contracts than ZeroCash. However, these solutions were generally built on the Ethereum platform and functioned via smart contracts, resulting in significant gas consumption and privacy vulnerabilities. After 2020, the emergence of DeFi drew attention to privacy protection in cross-chain exchanges. Phala ^17^, Raze Network ^18^, and Manta Network ^19^ focused on privacy protection in cross-chain DeFi based on the Substrate framework. They utilized zk-SNARKs to achieve end-to-end anonymity, high interoperability between chains, and a secure and user-friendly protocol. Nonetheless, with the increase in cryptocurrency-related illegal activities, governments worldwide have intensified their concerns regarding the regulation of privacy projects. Several privacy protection projects have been compelled to make improvements, including Zerocash and Tornado. The former had to incorporate regulatory keys during the 2019 Sapling update, while the latter faced sanctions and access restrictions in 2022. Consequently, research on regulatable privacy protection schemes currently offers broad application prospects.

Currently, there are multiple schemes available that can provide a certain degree of regulation while safeguarding user privacy. El Defrawy et al. proposed a scheme based on secure multiparty computation ^20^. This scheme ensures the traceability of user identity by distributed shares of the secret user identity to multiple servers. It requires reaching a threshold number of servers to recover the user identity. A scheme based on linkable group signatures provides both traceability of user identity and the auditability of transaction content ^21^. The linkable property enables other users to determine whether two transactions originate from the same sender, allowing for the identification of abnormal users. This scheme separates registration, auditing, and identity tracking operations among different entities, avoiding centralization. Li et al. introduced a regulatory scheme based on the Zerocash privacy protection scheme ^22^. In this scheme, regulatory authorities issue symmetric encryption keys to each regulated user. The users employ these keys to encrypt transaction-related information, while zero-knowledge proofs ensure consistency between encrypted information and transaction information. Regulatory authorities use their private keys to decrypt each ciphertext and obtain the transaction content of the regulated user. Lastly, centralized regulation often depends on third-party central nodes to conduct transaction regulation. For example, centralized mixing can be accomplished by employing mix servers as regulatory nodes. Group signatures can track the real signature user address through group administrators ^23^. Alternatively, users may be required to encrypt their corresponding privacy content before submitting it to the chain for review by regulators. The two-layer identity structure^24^ proposed by Hongbo Li and Tao Xie achieves decentralized e-commerce real-name supervision based on smart contracts, but does not support privacy protection for transaction information. Wang and Fu proposed RPTM^25^, which implements privacy-preserving task matching in blockchain-based crowdsourcing. RPTM can provide task matching services without compromising the privacy of task requesters and workers by utilizing a novel integer vector encryption scheme. Wang and Gao's proposal^26^ employs attribute-based encryption to achieve multi-level regulation on Bitcoin. However, the proposal only enables regulation of regular Bitcoin transactions and does not possess privacy protection capabilities. The multi-level regulation in this proposal allows different regulatory entities to oversee distinct user categories, with the ability to access users' true identities, the levels are not based on the content of regulation but rather on the range of user categories. Higher levels encompass a broader range of individuals, resulting in excessive concentration of power among high-level regulators and a lower degree of decentralization. The proposal^27^ put forward by Tianyu et al. achieves transaction regulation under privacy protection through the linkability of ring signatures. However, the comprehensiveness of regulatory content is severely lacking as it only reveals the sender's public key. Hyperledger Fabric^28^ utilizes Attribute-Based Encryption (ABE) to enforce access control rules in consortium blockchains, yet it lacks privacy protection features and only addresses user identity management in terms of regulation.

The details of comparison are shown in Table 1.

**Table 1:** Comparison of current regulatable privacy protection schemes.

Technical	Address	Value	Privacy	Efficiency	Regulatory	Projects
Centralized mixing	√	x	Low	High	Address	Mixcoin
Group signature	√	x	Low	Medium	Sender address	
Ring signature	√	√	Medium	Medium	Frequency	Monero
ZKP	√	√	High	Low	Address and value	ZeroCash
Range proof	x	√	Medium	Medium	Value range	Zether

The existing schemes for privacy protection with regulatory capabilities have certain limitations, and their suitability varies depending on the specific application scenario. These schemes commonly encounter the following issues: (1) limited regulatory content: most schemes can only access transaction addresses or statistical information, which fails to meet the comprehensive regulatory needs of most scenarios. (2) Privacy concerns: many schemes rely on technologies like coin mixing or ring signatures for regulation, but these technologies do not effectively safeguard user privacy. (3) Centralization of regulatory authority: the majority of schemes rely on a single third-party node for regulatory functions, leading to concentration of all regulatory information in one place. This increases the risk of information leakage and imposes a heavy workload on the regulatory entity.

## Preliminaries

### Notations

This paper presents a blockchain privacy protection scheme that supports multi-level regulation. It encompasses the definition and utilization of cyclic group, zero-knowledge proof, and various data structures. Some of the parameter symbols are shown in Table 2.

**Table 2:** Notations.

Notations	Descriptions	Notations	Descriptions
${G}_{n}$	Cyclic group	$\pi$	Zero-knowledge proof
*p*	Prime number	$cmt$	Value commitment
${e}$	Bilinear map	$sn$	Serial number
${Z}_{p}$	Integer group of order p	$r$	Random number
$\uplambda$	Initialization parameters	$(pk,sk)$	User address key pair
$C$	Circuit	$(PK,MK)$	ABE initial keys
$({pk}_{z},{vk}_{z})$	Zero-knowledge key pair	${SK}_{{u}_{i}}$	ABE private key
$\overrightarrow{x}$	Public input	${\text{CRF}}$	Hash function
$\overrightarrow{a}$	Private Input	${\text{COMM}}$	Commitment function

### Zero-knowledge proof

Zero-knowledge proof is a cryptographic technique that verifies data confidentiality without disclosing specific information. It proves the truth or falsehood of a proposition while maintaining privacy. The non-interactive zero-knowledge proof technology (zk-SNARKs)^29^ has three key characteristics: completeness, soundness, and zero-knowledge. Completeness: if a proposition is true, then an honest prover will with high probability be able to successfully pass the verification. Soundness: if a proposition is false, then a cheating prover with no information will only have a low probability of passing the verification. Zero-knowledge: apart from the truth or falsehood of the proposition, no other information is leaked. Zero-knowledge proof algorithms can be described as polynomial-time algorithms:

$${\prod }_{\rm Z}=({\text{Setup}},{\text{KeyGen}},{\text{Prove}},{\text{Verify}})$$

1. ${\text{Setup}}\left({1}^{\lambda }\right)\to {pp}_{Z}$. Given the security parameter λ, the algorithm performs an initialization operation to generate and output the public parameters ${pp}_{Z}=(p,e,{G}_{1},{G}_{2}, {G}_{T}, {P}_{1},{P}_{2},{F}_{p})$. Here, $p$ is a prime number; $e$ represents a bilinear map from ${G}_{1}\times {G}_{2}\to {G}_{T}$; ${G}_{1},{G}_{2}$, and ${G}_{T}$ are three cyclic groups of order $p$; ${P}_{1}$ and ${P}_{2}$ are the generators of ${G}_{1}$ and ${G}_{2}$, respectively; ${F}_{p}$ is a finite field. In zk-SNARKs, all other algorithms take ${{\text{pp}}}_{Z}$ as the default input for public parameters.
2. ${\text{KeyGen}}\left({\text{C}}\right)\to ({pk}_{z},{vk}_{z})$. Given a circuit ${\text{C}}$, this algorithm utilizes the public parameters ${{\text{pp}}}_{Z}$ to generate a key pair $({pk}_{z},{vk}_{z})$, where ${pk}_{z}$ is the proving key used for generating zero-knowledge proofs, and ${vk}_{z}$ is the verification key used for verifying zero-knowledge proofs.
3. ${\text{Prove}}\left({pk}_{z},\overrightarrow{x},\overrightarrow{w}\right)\to \pi$. This algorithm is used to generate a zero-knowledge proof $\pi$. In the input parameters,$\overrightarrow{x}$ represents the input of circuit ${\text{C}}$, which is a publicly declared state; $\overrightarrow{w}$ represents the auxiliary input of circuit ${\text{C}}$, which is a private evidence; $\pi$ is the zero-knowledge proof that demonstrates the correspondence between $\overrightarrow{x}$ and $\overrightarrow{w}$ satisfying the construction of circuit ${\text{C}}$. It should be noted that $\overrightarrow{x}$ and $\pi$ are publicly available and visible to anyone.
4. ${\text{Verify}}\left({vk}_{z},\overrightarrow{x},\pi \right)\to b$. With the use of this algorithm, anyone can check and verify the validity of zero-knowledge proofs. If the zero-knowledge proof is successfully verified, the algorithm outputs $b=1$; otherwise, it outputs $b=0$ to indicate the failure of verification.

**Figure 1:**
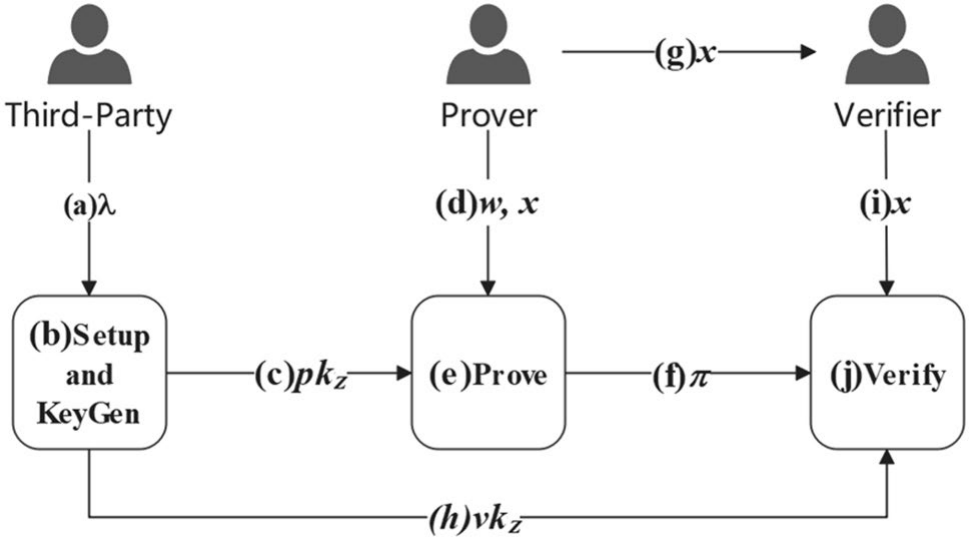
Zero-knowledge proof algorithm.

The workflow of zero knowledge proof is shown in Fig. 1.

### Attribute-based encryption

Attribute-based encryption (ABE) is a one-to-many access control method for public key encryption^30^. The data owner begins by defining a security policy for their data. Subsequently, a key authority converts this policy into encryption keys. Only users who satisfy the conditions specified by the policy can decrypt the data successfully. This approach strengthens data security and privacy. The algorithmic details are as follows:

$${\prod }_{A}=({\text{Setup}},{\text{KeyGen}},{{\text{Encrypt}}}_{ABE},{{\text{Decrypt}}}_{ABE})$$

1. ${\text{Setup}}\left({1}^{\lambda }\right)\to (PK,MK)$. Given the security parameter λ as input, the algorithm outputs the initial keys $(PK,MK)$ for the system.
2. ${\text{KeyGen}}(MK,{A}_{{u}_{i}})\to {SK}_{{u}_{i}}$. The key authority runs this algorithm to generate a private decryption key ${SK}_{{u}_{i}}$, corresponding to the attributes ${A}_{{u}_{i}}$ possessed by the user. The private keys are generated by a random algorithm executed by the key authority, creating a private key for each attribute tree in the attribute domain.
3. ${{\text{Encrypt}}}_{ABE}(PK,m,\mathcal{T})\to CT$. The ciphertext $CT$ is generated by a random algorithm executed by the data owner. This algorithm takes the message $m$ to be encrypted, the access policy $\mathcal{T}$ defined by a set of attributes, and the public key $PK$ as inputs.
4. ${{\text{Decrypt}}}_{ABE}({SK}_{{u}_{i}},CT,PK)\to m$. A user who possesses the attributes satisfying the access policy uses this algorithm with the corresponding key ${SK}_{{u}_{i}}$ to decrypt the ciphertext $CT$ and recover the message $m$.

## Our scheme

We will describe the principles and operational steps of this approach to achieving privacy protection and multi-level regulation in this section. Section “Data structure”, provides a detailed description of the data structures involved in this approach, including their mathematical symbols and key roles. Section “Privacy model” presents the workflow of the privacy protection model, highlighting the application of zk-SNARK in the approach and explaining the functions and important parameters of the main algorithms. Lastly, in "Multi-level regulatory model", we specifically focus on how multi-level regulation operates in conjunction with the privacy protection model, examining the application of attribute-based encryption (ABE) in regulation, as well as outlining the specific responsibilities and tasks of regulators at different levels.

### Data structure

The commitment of balance is defined as $cmt={\text{COMM}}(addr,value,sn,r)$, which is stored in the account as the user's balance. After each transaction, the user updates the commitment and submits it for verification by miners.

The commitment of transfer is defined as ${cmt}_{v}={\text{COMM}}({addr}_{A},v,{pk}_{B}, {sn}_{v},{sn}_{A},{r}_{v})$. It is related to the transfer information in the Send transaction and its compliance is ensured through zero-knowledge proofs.

The serial number is defined as $sn={\text{CRF}}(sk,r)$. The serial number $sn$ accompanies each balance commitment and transfer commitment. It is generated by the random number $r$ and the user's private key $sk$, ensuring that $sn$ must be generated by the initiator of the corresponding transaction.

Zero-knowledge balance $zk\_balance=\{cmt, addr,value,sn,r\}$. $zk\_balance$ is a set of parameters related to the user's account balance.

Commitment set $CMTSet$. It stores the set of ${cmt}_{v}$ from Send transactions within each block.

Serial number set $SNSet$. It is responsible for storing all transfer commitments ${sn}_{A}$. Whenever a miner verifies the validity of a transaction, they need to check if ${sn}_{A}$ has appeared in $SNSet$. This method can help resist double-spending attacks.

### Privacy model

**Figure 2:**
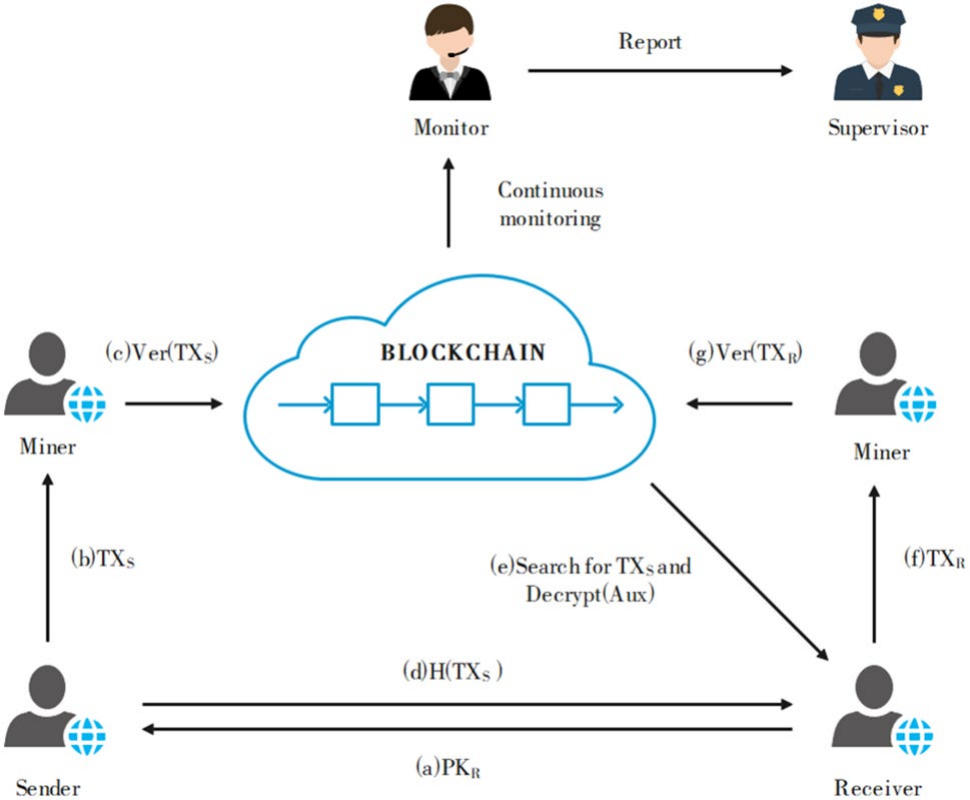
Workflow of privacy transactions.

Inspired by BlockMaze^16^, this solution utilizes zk-SNARKs to achieve unlinkability of transaction addresses and transaction content privacy in the account model. It employs commitments to protect account balances, transfer values, and the correspondence between senders and receivers. The solution incorporates a two-step transfer mechanism: senders first send funds to the blockchain, and then receivers deposit the funds from the blockchain. This mechanism safeguards the correlation between senders and receivers. To protect balance information on the public ledger, only the commitment $cmt$ of the corresponding value is recorded through $zk\_balance$. During transfers, the receiver's address is not included in the transaction. Instead, the receiver receives a hash value $h$ of the transaction, concealing the sender's address. The receiver can use $h$ to retrieve the corresponding transaction on the chain.When depositing funds, the receiver places the commitment ${cmt}_{v}$ of the transfer value on a leaf node of a Merkle Patricia Trie (MPT) tree. The root rt of the tree is utilized in the zero-knowledge proof to hide the commitment of the transfer value and the sender's address. Both the sending and receiving processes ensure security and privacy through zero-knowledge proofs using zk-SNARKs. Blockchain validators validate transfer operations by verifying the zero-knowledge proofs, without gaining access to the transfer value or the relationship between senders and receivers. The workflow of privacy model is shown in Fig. 2.

The algorithm descriptions involved in the entire privacy transaction are as follows:

${\text{Setup}}({1}^{\lambda })\to {\text{pp}}$: Given a security parameter λ, this algorithm generates a public system parameter list ${\text{pp}}$, which is publicly accessible to anyone. It is important to note that the ${\text{Setup}}$ algorithm can only be executed by a trusted third party and should be executed once.
${\text{CreateAccount}}\left({\text{pp}},ID\right)\to \{addr,(pk,sk)\}$: Given the public parameter list ${\text{pp}}$, this algorithm creates an account address for the user and generates a key pair $(pk,sk)$. The private key $sk$ is used to access private data and decrypt ciphertext data in transactions, while the public key $pk$ is used to encrypt shared transaction parameters. The account address is used for sending and receiving transfer funds. At the same time, the public key $pk$ is generated and issued to the user by a key management organization, and it is bound to the user's real identity information $ID$.
${\text{Send}}\left({zk\_balance}_{A},{sk}_{A},{pk}_{B},v\right)\to \{{zk\_balance}_{A}^{*},{tx}_{S}\}$: This algorithm allows sender A to send zero-knowledge value to receiver B. Given the current zero-knowledge balance ${zk\_balance}_{A}$ of account A, the sender's account private key ${sk}_{A}$, the receiver's account public key ${pk}_{B}$, and the plaintext value $v$ to be transferred as zero-knowledge value, account A can use this algorithm to update its zero-knowledge balance ${zk\_balance}_{A}^{*}$ and generate transaction ${tx}_{S}$.
(4)${\text{Receive}}\left({zk\_balance}_{B},{{pk}_{B},sk}_{B},{{\text{h}}}_{{tx}_{S}}\right)\to \{{zk\_balance}_{B}^{*},{tx}_{D}\}$: This algorithm allows receiver B to check and store the received value in their account. Given the current ledger, public parameters, account key pair (${pk}_{B},{sk}_{B}$), the hash value ${{\text{h}}}_{{tx}_{S}}$ of transaction ${tx}_{S}$, and the current zero-knowledge balance ${zk\_balance}_{B}$ of account B, receiver B calls the ${\text{Receive}}$ algorithm to receive and deposit the payment, obtaining a new zero-knowledge balance ${zk\_balance}_{B}^{*}$ and generating transaction ${tx}_{D}$.
${\text{Verify}}\left(tx\right)\to b$: Given the current ledger and transaction $tx$, the miners use this algorithm to check the validity of all zero-knowledge transactions. If the $tx$ is valid, the algorithm outputs $b=1$; otherwise, it outputs $b=0$. Miners (or nodes maintaining the blockchain) are responsible for verifying all transactions and updating the state of the relevant accounts.

### Multi-level regulatory model

We propose a multi-level regulatory model based on existing privacy models. The model operates as follows: observers monitor the ledger for abnormal fluctuations in transaction frequency, allowing them to detect suspicious transactions or accounts. They report these findings to higher-level regulators. Third-level regulators are responsible for the disclosure of specific transaction information. Attribute-based encryption (ABE) and zero-knowledge Succinct Non-interactive Arguments of Knowledge (zk-SNARKs) are utilized to support the entire process. The regulatory model of this scheme possesses the following characteristics:

1. ABE is used to selectively disclose privacy information in transactions, allowing different levels of regulators to access specific information. This decentralizes regulatory work to some extent. Moreover, the one-to-many nature of ABE enables each regulator to possess a unique key, reducing the management cost of regulatory keys.
2. By leveraging the features of send and receive transactions, incentive measures can be implemented to encourage active participation in basic transaction monitoring by ordinary users or miners. This reduces the workload of dedicated regulators and facilitates the detection of abnormal transaction behavior.
3. Relevant government departments serve as trusted third parties, fulfilling roles such as user identity verification (Know Your Customer, KYC), attribute key management for different levels of roles, and acting as the highest authority for transaction regulation. This ensures compliance with mandatory regulatory requirements imposed by various countries on blockchain privacy projects.
4. Zero-knowledge proofs are utilized throughout the process to maintain consistency between regulatory information and transaction information. This prevents the use of false information by transacting parties to evade monitoring.

**Figure 3:**
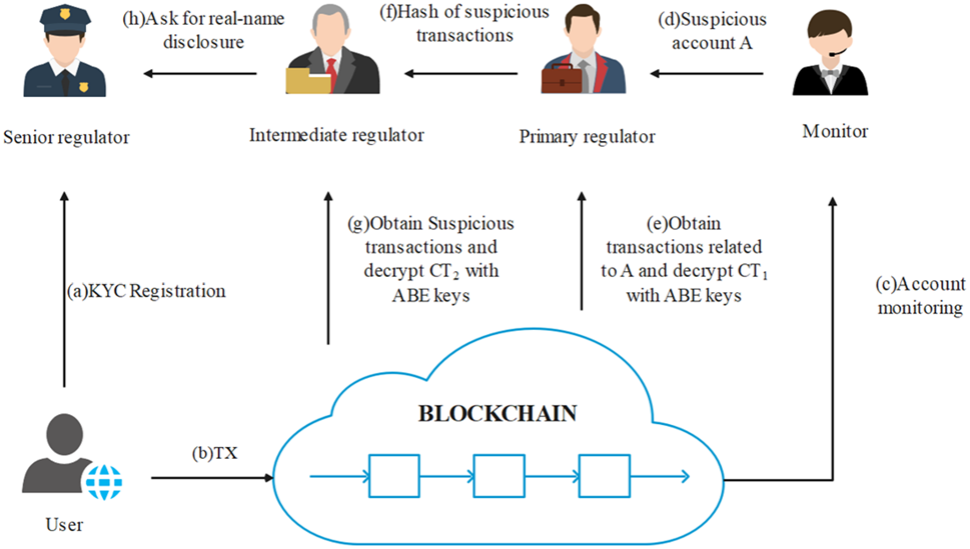
Workflow of multi-level regulatory.

The workflow of multi-level regulation is shown in Fig. 3.

Roles in the regulatory scheme can be divided as follows:

### User

The main role involved in privacy transactions, including the sender and receiver of the transaction. Their tasks include generating send and receive transactions, broadcasting them to the blockchain, and updating zero-knowledge balances in their accounts. Users also need to encrypt different levels of privacy information using public keys of different attributes and include them in the transaction on the blockchain. Additionally, they generate corresponding zero-knowledge proofs for miners to verify.

### Miner

Miners are responsible for verifying, packaging, and broadcasting processes related to privacy transactions. They must validate the legitimacy of the transaction without knowing its value or addresses involved. The specific process is as follows: first, they verify whether ${cmt}_{v}$ and ${sn}_{v}$ exist in $CMTSet$ and $SNSet$, respectively, to prevent double-spending attacks. Then they verify the correctness of the zero-knowledge proof corresponding to the transaction to ensure that the transaction value is within the correct range. Finally, they verify the correctness of the zero-knowledge proof corresponding to the regulatory ciphertext to ensure consistency between the regulatory information and the transaction information.

### Monitor

The main role of the monitor is to observe fluctuations in user transaction frequency to identify abnormal accounts. This role can be performed by ordinary users or miners. By tracking the frequency of send and receive transactions associated with a specific address, monitors can detect suspicious behavior, such as a significant increase in transaction volume within a specific time period. Monitors should report these findings to higher-level regulators, and valuable information reported may result in partial rewards.

### Primary regulator

The primary regulator's main task is to trace the addresses of both parties involved in the transaction. Employees hired by virtual service providers usually perform this role. They use their attribute public keys to decrypt relevant fields in send transactions, obtaining the recipient's public key. Similarly, they decrypt relevant fields in receive transactions to obtain the sender's public key. This enables the establishment of a mapping relationship between the addresses of the transaction parties, completing the tracking of transaction addresses.

### Intermediate regulator

The main task of the intermediate regulator is to query the specific value of privacy transactions. Administrators of virtual service providers usually perform this role. They use their attribute public keys to decrypt relevant fields in send and receive transactions, obtaining the transaction value and completing the tracking of the content of privacy transactions.

### Senior regulator

The senior regulator's main tasks include user registration, distribution of regulatory keys, and providing real-name regulation for illegal transactions. This role is typically undertaken by relevant administrative departments. They generate key pairs $(pk,sk)$ associated with users' real identities as the public and private keys of the transaction account. Additionally, they issue attribute keys to regulators at all levels to enable real-name tracking of illegal users.

From the functional allocation of the regulatory model, it is evident that regulators at different levels can only access transaction-related information such as $addr$ and $value$. They cannot access secret parameters like $sk, {sn}_{v},$ or ${r}_{v}$, which are required to generate spending proofs ${\pi }_{s}$. As a result, all regulators are unable to impersonate traders and spend the balances in their accounts, ensuring the security of the transaction model.

## The protocol description

Building upon BlockMaze^16^, we have amended the privacy protocol to enhance its support for multi-level regulation. Within the Setup, sections for ABE algorithm initialization and key distribution have been incorporated. In the Send and Receive algorithms, the generation of regulatory ciphertext for transaction value and address is now based on the regulation permission tree. Additionally, a circuit has been included in the zero-knowledge proof to demonstrate the consistency between the regulatory ciphertext and the transaction value. Finally, the Regulate algorithm delineates the regulatory actions initiated by regulators of different levels for private transactions, demonstrating the distinctions between regulation permissions and contents. The following provides a detailed description of each algorithm.

### Setup

${\text{Setup}}$ Is an algorithm used to generate a system's public parameter list. In order to construct zero-knowledge transactions, it is necessary to design specific circuits, denoted as $C$, to ensure that the state of the accounts before and after executing the algorithmic operations, as well as the constructed transactions, are all valid and legal. Key pairs are generated for both proving and verifying these circuits. It is important to note that this algorithm is executed only once by a trusted third party. The detailed is as follows:
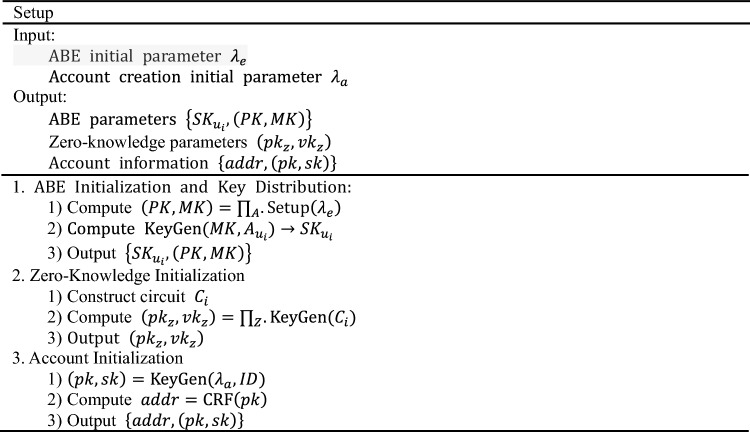


### Send

The Send transaction is used by the sender to transfer funds and generate transaction ${tx}_{S}$. After generating the ${tx}_{S}$ transaction, account A informs account B offline of the transaction hash value ${{\text{h}}}_{{tx}_{S}}={\text{CRF}}({tx}_{S})$ for retrieval and parsing of ${tx}_{S}$, enabling subsequent Receive operations to construct ${tx}_{D}$ for deposit. Once ${tx}_{S}$ is agreed upon by miners and recorded on the blockchain, the state of account A undergoes the following changes: prior to executing the Send algorithm, the state of account A is $\left\{pt\_balance,cmt\right\}$; after executing the Send algorithm, the state of account A is $\{pt\_balance,{cmt}^{*}\}$. The detail is as follows:
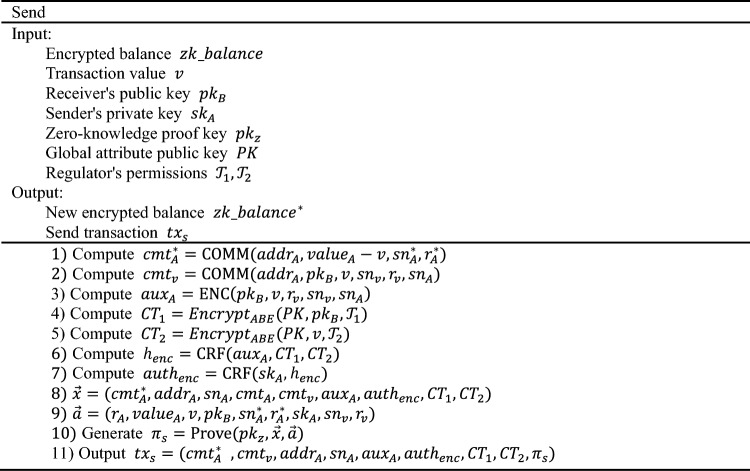


The ${\pi }_{s}$ is the core content of the Send transaction, which can prove the following:

1. ${value}_{A}>=v$The value v in the Send transaction must be less than or equal to the balance ${value}_{A}$ of account A to prevent users from spending more than the available balance.
2. ${sn}_{A}={\text{CRF}}\left({sk}_{A},{r}_{A}\right),{sn}_{A}^{*}={\text{CRF}}\left({sk}_{A},{r}_{A}^{*}\right),{sn}_{v}={\text{CRF}}({sk}_{A},{r}_{v})$ The serial numbers $sn$ used in the Send transaction are correctly generated and bound to the private key ${sk}_{A}$ of account A, ensuring they cannot be forged.
3. ${cmt}_{A}=\mathrm{C}\mathrm{O}\mathrm{M}\mathrm{M}\left({addr}_{A},{value}_{A},{sn}_{A},{r}_{A}\right),{cmt}_{A}^{\mathrm{*}}=\mathrm{C}\mathrm{O}\mathrm{M}\mathrm{M}\left({addr}_{A},{value}_{A}-v,{sn}_{A}^{\mathrm{*}},{r}_{A}^{\mathrm{*}}\right),\, {cmt}_{v}={\text{COMM}}({addr}_{A}, {pk}_{B},v,{sn}_{v},{r}_{v},{sn}_{A})$. The balance commitments $cmt$ used in the Send transaction are correctly generated and bound to the account's address $addr$. Additionally, the binding relationship between ${sn}_{A}$ and ${r}_{A}$ ensures that they cannot be forged.
4. ${auth}_{enc}=\mathrm{CRF}\left({sk}_{A},\mathrm{h}_{enc}\right)$. The signature ${auth}_{enc}$ is a signature about ${h}_{enc}$, proving that the ciphertext ${aux}_{A},{CT}_{1},$ and ${CT}_{2}$ have not been modified.

### Receive

The Receive transaction is used by the recipient to receive funds. The recipient receives the off-chain ${h}_{tx}={\text{CRF}}({tx}_{send})$ sent by the sender and retrieves the corresponding transaction on the blockchain. During this process, there is no direct interaction between the recipient and the sender. When receiving funds, it is not advisable to directly disclose ${cmt}_{v}$ as part of the $statement$, as it would link back to the sending transaction ${tx}_{send}$. Therefore, ${cmt}_{v}$ is used as a leaf node to construct a Merkle Tree, with its root $rt$ as part of the $statement$. The relationship between $rt$ and ${cmt}_{v}$ is then proved. The detail is as follows:
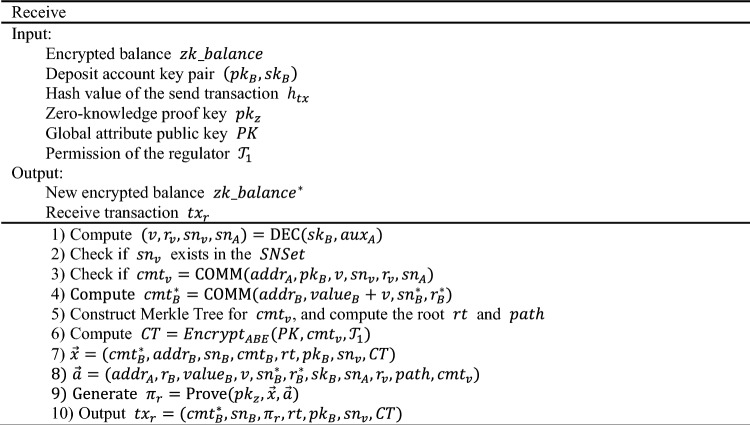


The ${\pi }_{r}$ is the core content of the Receive transaction, which can prove the following:

1. ${sn}_{B}={\text{CRF}}\left({sk}_{B},{r}_{B}\right),{sn}_{B}^{*}={\text{CRF}}\left({sk}_{B},{r}_{B}^{*}\right)$. The serial numbers $sn$ involved in the Receive transaction are correctly generated and bound to the private key ${sk}_{B}$ of account B, making them unable to be forged.
2. $${cmt}_{B}={\text{COMM}}\left({addr}_{B},{value}_{B},{sn}_{B},{r}_{B}\right),{cmt}_{B}^{*}={\text{COMM}}\left({addr}_{B},{value}_{B}+v,{sn}_{B}^{*},{r}_{B}^{*}\right),$$
  ${cmt}_{v}={\text{COMM}}({addr}_{A}, {pk}_{B},v,{sn}_{v},{r}_{v},{sn}_{A})$. The balance commitments $cmt$ involved in the Receive transaction are correctly generated and bound to the account's address $addr$. Moreover, ${sn}_{B}$ is bound to ${r}_{B}$, preventing them from being forged.
3. $rt=path({cmt}_{v})$. $rt$ is the Merkle root of the Merkle tree $CMTSet$ concerning the transfer commitment ${cmt}_{v}$. It can prove that ${cmt}_{v}$ has indeed appeared in $CMTSet$ and is related to the current $rt$ generation.

### Verify

The algorithm checks and verifies all zero-knowledge transactions. Once these transactions are packaged into candidate blocks, miners will examine each transaction to confirm whether the relevant account information (e.g., serial numbers of balance commitments and fund transfer commitments) has been previously disclosed and if the Merkle roots in the transaction are valid. If all the aforementioned checks pass, miners will proceed with the following operations: (1) update the zero-knowledge balance commitments of the relevant accounts in the transaction, i.e., update ${cmt}_{A}$ to ${cmt}_{A}^{*}$; (2) append the disclosed serial numbers (such as ${sn}_{A}$, ${sn}_{B}$, ${sn}_{v}$) in the transaction to SNSet to prevent double-spending attacks; (3) append the fund transfer commitment (such as ${cmt}_{v}$) to $CMSet$ in the block, awaiting the recipient to make a deposit. The detailed process is as follows:
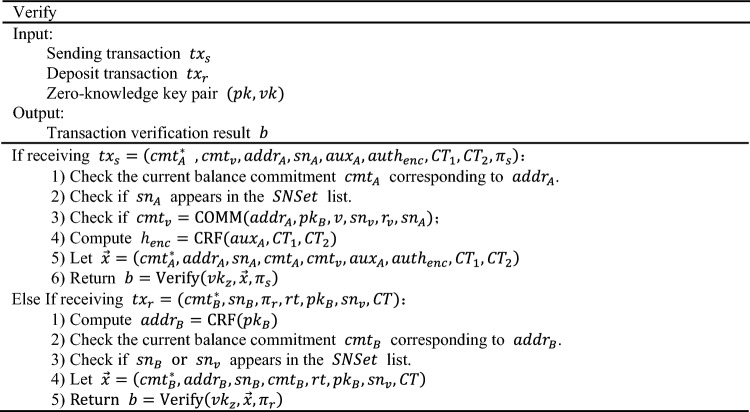


The verification includes the following aspects: (1) verify if the corresponding sequence number $sn$ is in the spent list for each transaction. (2) For receive transactions, verify if ${cmt}_{v}$ and its corresponding $path$ can generate the root $rt$. (3) Verify the zero-knowledge proof corresponding to each transaction. (4) Miners also need to update the zero-knowledge balance and sequence number list, and add ${cmt}_{v}$ to the block.

### Regulate

The algorithm includes methods for different levels of regulators to monitor transaction information. Monitors can observe the transaction frequency of accounts from the public ledger. If an abnormal frequency is identified within a certain time period, the corresponding account address can be provided to higher-level regulators. The primary regulator can decrypt ${CT}_{1}$ using attribute keys to obtain the addresses of the transacting parties, while the intermediate regulator can decrypt ${CT}_{2}$ using attribute keys to obtain the transaction value $v$. Finally, after comprehensive analysis, if it is found that an account is involved in illegal transaction activities, the address can be submitted to the senior regulator to complete the real-name tracking of the account user. The detailed process is as follows:
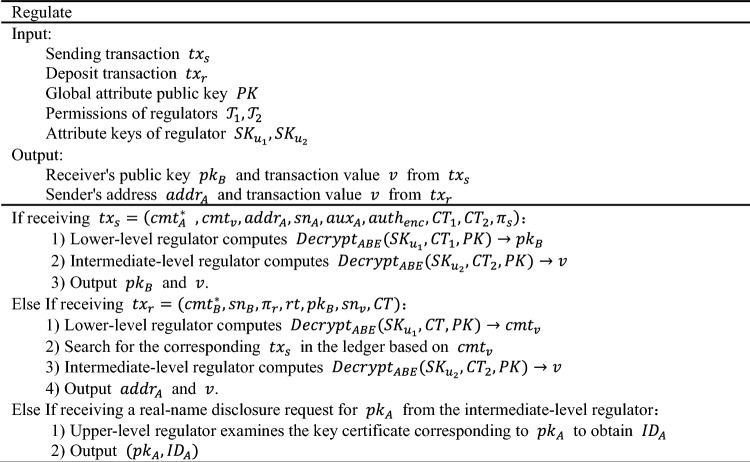


## Security analysis

According to the security model defined by ZeroCash, this scheme satisfies ledger indistinguishability, transaction unlinkability, transaction non-malleability, and balance conservation. The specific analysis is as follows:

1. Ledger indistinguishability Ledger indistinguishability means that if an adversary can only obtain information publicly available in the ledger but cannot access any new useful information, then the ledger is considered indistinguishable. In the ledger, only the balance commitment $cmt$ of an account is publicly disclosed. Due to the one-way property of the cryptographic random function (CRF), it is impossible to derive the plaintext balance ${value}_{A}$ from $cmt$. Therefore, the account balance is considered private. The transfer value $v$ in transaction ${tx}_{s}$ is also hidden in the transfer commitment ${cmt}_{v}$ and the transmitted ciphertext ${aux}_{A}$. Under the security of encryption ENC, only the owner of ${sk}_{B}$ can obtain the plaintext value. The updated ${cmt}_{B}^{*}$ in transaction ${tx}_{r}$ cannot reveal the receiver's plaintext balance or obtain the corresponding information from ${tx}_{s}$. Overall, under the security of CRF and ENC, the adversary cannot obtain any useful information beyond what is publicly available. Therefore, we consider this scheme to have ledger indistinguishability.
2. Transaction unlinkability. Transaction unlinkability means that the transfer relationship between sender and receiver is not disclosed during fund transfer. First, let's analyze the data structure of the send transaction, ${tx}_{s}=\left({cmt}_{A}^{*},{cmt}_{v}, {addr}_{A}, {sn}_{A}, {aux}_{A}, {auth}_{enc},{{CT}_{1},{CT}_{2},\pi }_{s}\right)$. From the publicly available content, we can obtain the sender's address ${addr}_{A}$ and the new account balance ${cmt}_{A}^{*}$ related to the transaction address. Information related to the receiver's address is hidden in ${cmt}_{v}$, ${aux}_{A}$, ${CT}_{1}$, and ${\pi }_{s}$. As long as the security of CRF, attribute-based encryption (ABE), and zk-SNARKs is ensured, the probability of the adversary obtaining receiver-related information from ${tx}_{s}$ can be considered negligible. Next, let's consider the receive transaction, ${tx}_{r}=({cmt}_{B}^{*}, {sn}_{B}, {\pi }_{r}, rt,{pk}_{B}, {sn}_{v},CT)$. From this information, we know that the receiver's public key ${pk}_{B}$ and the new account balance ${cmt}_{B}^{*}$ are publicly disclosed. Information related to the sender is hidden in $CT$ and $rt$. As long as the security of ABE and Merkle-Tree is ensured, the probability of the adversary obtaining sender-related information from ${tx}_{r}$ can be considered negligible. Therefore, it can be proven that the adversary cannot obtain receiver-related information from ${tx}_{s}$ or sender-related information from ${tx}_{r}$, thus satisfying transaction unlinkability.
3. Transaction non-malleability. Transaction non-malleability refers to the property that if an adversary cannot generate new valid transactions using the publicly available information, then the transactions are non-malleable. First, let's analyze the generation of zero-knowledge proofs in the ${tx}_{s}$. The public information ${tx}_{s}=\left({cmt}_{A}^{*},{cmt}_{v}, {addr}_{A}, {sn}_{A}, {aux}_{A}, {auth}_{enc},{{CT}_{1},{CT}_{2},\pi }_{s}\right)$ and the private witness $\overrightarrow{a}=({r}_{A}, {value}_{A}, v,{pk}_{B},{sn}_{A}^{*},{r}_{A}^{*}, {sk}_{A}, {sn}_{v},{r}_{v})$ are publicly known and secret, respectively. If the adversary wants to construct a new valid ${tx}_{s}$, they would need to be able to generate new sequence numbers ${sn}_{A}^{*}$ and ${sn}_{v}$ using the public data, as well as generate the corresponding zero-knowledge proof ${\pi }_{s}$. However, these operations cannot be performed without knowledge of the sender's private key ${sk}_{A}$. Therefore, as long as the security of CRF and zk-SNARKs is ensured, the probability of an adversary constructing a new valid ${tx}_{s}$ can be considered negligible. Similarly, in the ${tx}_{r}$, the information ${tx}_{r}=({cmt}_{B}^{*}, {sn}_{B}, {\pi }_{r}, rt,{pk}_{B}, {sn}_{v},CT)$ and the witness $\overrightarrow{a}=({addr}_{A}, {r}_{B}, {value}_{B}, v, {sn}_{B}^{*},{r}_{B}^{*}, {sk}_{B},{sn}_{A},{r}_{v},path, {cmt}_{v})$ are publicly known and secret, respectively. If the adversary wants to construct a new valid ${tx}_{r}$, they would need to be able to generate new sequence numbers ${sn}_{B}^{*}$ as well as decrypt certain information such as ${r}_{v}$ and ${cmt}_{v}$ using ${aux}_{A}$. However, these operations cannot be performed without knowledge of the receiver's private key ${sk}_{B}$. Therefore, as long as the security of CRF and zk-SNARKs is ensured, the probability of an adversary constructing a new valid ${tx}_{r}$ can be considered negligible. Hence, it can be proven that adversaries cannot construct new ${tx}_{s}$ and ${tx}_{r}$ from the publicly available information, and therefore, we consider our scheme to satisfy transaction non-malleability.
4. Balance conservation. Balance conservation refers to the property that if an adversary can only spend from their own account balance and cannot spend nonexistent values, then the balance on the ledger is conserved. We analyze the data structure of the Send transaction. In ${tx}_{s}$, ${cmt}_{A}^{*}$ represents the new account balance after spending and is part of the public parameters generated by ${\pi }_{s}$. It is generated as ${cmt}_{A}^{*}={\text{COMM}}({addr}_{A},{value}_{A}-v,{sn}_{A}^{*},{r}_{A}^{*})$, and ${\pi }_{s}$ can prove that ${value}_{A}>=v$. If the adversary wants to overspend, it would result in ${value}_{A}<v$, which contradicts what ${\pi }_{s}$ is intended to prove. Therefore, it can be proven that adversaries cannot generate ${\pi }_{s}$ for overspending, and thus we consider our scheme to satisfy balance conservation.

## Performance analysis

This section outlines the specific implementation process of the proposed scheme. The scheme is built upon the account model blockchain Ethereum and makes use of core technologies such as zero-knowledge proofs (libsnark) and attribute-based encryption (openabe). These technologies modularize the zero-knowledge circuits designed in this paper. Additionally, an experimental procedure is devised to evaluate the scheme's performance. The experiment is conducted on Ubuntu 22.04, utilizing an Intel(R) Core(TM) i7-11800H @ 2.30 GHz CPU.

To assess the effectiveness of our scheme in terms of privacy protection and regulatory capability, we compare it with other similar schemes. Firstly, we consider the privacy performance of different types of blockchain transactions, focusing on transaction address privacy and transaction value privacy. Additionally, we evaluate the comprehensiveness of regulatory coverage and, based on that, examine the level of centralization among the regulatory parties. The specific comparison details can be found in Table 3.

**Table 3:** Comparison of privacy-preserving schemes with regulation compliance.

Technology	Transaction model	Address	Value	KYC	Regulatory content	Regulatory role
Mixcoin	UTXO	√	x	x	Addresses	Exchange
ZeroCash	UTXO	√	x	x	Sender's address	Single party
Monero	UTXO	√	√	x	Transaction frequency	All users
CP-HABE Scheme	UTXO	x	x	√	Identity of users	Multi-level parties
BlockMaze	Account	√	√	x	Transaction frequency	All users
Hyperledger Fabric	Account	x	x	√	Identity of users	Single party
Zether	Account	x	√	x	Range of value	All users
Our scheme	Account	√	√	√	Addresses and values	Multi-level parties

According to Table 3, Mixcoin, ZeroCash, Monero, and CP-HABE Scheme are all based on the UTXO model of Bitcoin, which lacks support for smart contracts and has limited application scenarios. Mixcoin, CP-HABE Scheme, Hyperledger Fabric, and Zether do not provide comprehensive privacy protection for on-chain transactions, typically only protecting either transaction addresses or values, or even lacking privacy protection entirely. While BlockMaze offers strong privacy features and supports smart contracts, it does not consider the traceability of privacy transactions. Mixcoin, ZeroCash, and Monero implement transaction address regulation, while CP-HABE Scheme and Hyperledger Fabric implement user identity regulation, and Zether implements range auditing of transaction values. However, the scope of regulation is relatively limited, with regulatory power too centralized in a single regulator. Overall, our proposed solution achieves privacy protection of transaction addresses and values based on the account model, while simultaneously implementing multi-level regulation on top of privacy protection. It can regulate transaction frequency, address, value, and real identities, with regulatory power dispersed among regulators of different levels, avoiding centralization of regulation. Therefore, our solution has functional advantages compared to existing solutions.

To evaluate the efficiency of our scheme, we analyze the time and space consumption of the core algorithm. Generating zero-knowledge proofs for send and receive transactions incurs a significant computational cost. However, it is worth noting that these proofs are created by users off-chain. Therefore, the computational cost of verification (Verify) becomes a key factor in our evaluation. Another aspect to consider is the space consumption during the Setup process, primarily attributed to the generation of public parameters. The storage size of proof keys and verification keys generated by third parties also plays a crucial role. The performance of the privacy algorithm of the scheme is shown in Table 4.

**Table 4:** Performance of privacy algorithms.

Send	Setup time	25.911 s	Proof size	127.38 B
	${pk}_{z}$ size	36.94 MB	Prove time	7.468 s
	${vk}_{z}$ size	358.5 B	Verify time	5.417 ms
Receive	Setup time	55.328 s	Proof size	127.38 B
	${pk}_{z}$ size	74.39 MB	Prove time	17.844 s
	${vk}_{z}$ size	398.38 B	Verify time	4.589 ms
Regulate	Setup time	4.767 ms	Keygen time	13.549 ms
	Encrypt time	11.525 ms	Decrypt time	4.09 ms
	CT size	168 B		

Due to significant disparities in efficiency caused by different privacy protection approaches, we have chosen Zerocash and BlockMaze, which follow a similar technical path as our solution, for efficiency comparison. The primary metrics considered are the generation time and storage space consumption of zero-knowledge proofs within the privacy algorithms, followed by the crucial on-chain verification time. Please refer to Table 5 for specific comparative data.

**Table 5:** Performance comparison of privacy algorithms.

Our scheme		BlockMaze		ZeroCash	
Send	Prove time 7.468 s	Send	Prove time 6.133 s	Mint	Prove time 1 µs
	Size 127.38 B		Size 127.38 B		Size 72 B
Receive	Prove time 17.844 s	Deposit	Prove time 13.261 s	Pour	Prove time 94.53 s
	Size 127.38 B		Size 127.38 B		Size 1004B
Verify	Average Time 5.003 ms	Verify	Average Time 4.427 ms	Verify	Pour Time 7.51 ms
Regulate	Time 4.09 ms			Receive	Time 1.92 ms
	Size 168 B				

As indicated in Table 5, our proposed scheme exhibits higher time consumption in proof generation and verification compared to BlockMaze. However, the disparity in on-chain verification time, which is of greater significance, remains within acceptable limits. In contrast to ZeroCash, our scheme demonstrates a substantial advantage in terms of generation time. Although our solution incurs slightly higher total verification time, the difference is minimal.

### Discussion

Based on the experimental results, it is evident that our scheme, when implementing multi-level regulation, has slightly lower efficiency compared to existing solutions. Specifically, introducing consistency proofs for ABE ciphertext during the Send transaction generation process has led to a more complex zero-knowledge proof circuit. However, this has not significantly increased the transaction verification time, thereby having minimal impact on on-chain verification overall, which falls within an acceptable range. Nevertheless, there are still several areas that require improvement in our proposed solution.

1. The zero-knowledge proof algorithm still relies on a trusted initialization setup to obtain proof keys and verification keys for privacy transaction proof generation. The security of the proof algorithm is susceptible to the leakage of intermediate parameters during this process. Additionally, reliance on a trusted third party raises operational costs and enhances the level of centralization in our solution. In the future, it would be advantageous to explore zero-knowledge proof algorithms that do not necessitate trusted setups
2. The senior regulators hold significant power within the regulatory roles, as they have control over the generation and distribution of ABE keys. They also possess the capability to hold accountable users suspected of illegal transactions or regulators engaging in non-compliant operations. It is essential to distribute or oversee the power of these superior regulators. One potential approach could involve establishing a decentralized key distribution organization composed of multiple government agencies that distribute authority among different entities.
3. The design of the multi-level regulatory structure is still relatively basic. Currently, non-compliant actions by ordinary regulators can only be addressed through real-name accountability or deducting their security deposits by service providers. There is a lack of corresponding technical means. The attribute design of regulators' ABE keys is not comprehensive enough, and it can be gradually improved in the subsequent application process.
4. The efficiency of the zero-knowledge proof algorithm still requires enhancement, particularly concerning the use of the SHA-256 algorithm in commitment functions. The excessive number of multiplication gates inserted into the zero-knowledge circuit results in a high computational cost for generating Merkle Tree path proofs. To address this, future exploration can focus on optimizing the HASH algorithm and Merkle Tree to improve performance in the zero-knowledge proof algorithm.

## Conclusions

In recent years, blockchain technology had experienced continuous development, leading to its practical application. However, traditional blockchains still face privacy leakage issues that need to be addressed. While previous methods had focused on enhancing user privacy, there had been limited exploration of regulatory methods for the blockchain. This paper introduces a blockchain ledger privacy protection scheme that supports multi-level regulation using zero-knowledge proofs (zk-SNARKs) and attribute-based encryption (ABE). The scheme ensures user privacy while allowing for transaction public verification. It enables various levels of regulators to trace user transaction privacy, ensuring comprehensive regulatory coverage. We also discuss practical issues such as regulatability, security, and privacy in detail. Our analysis demonstrates that our scheme provides sufficient security, stronger anonymity compared to similar schemes, and avoids concentration of regulatory power. Experimental results indicate that our scheme performs well in terms of verification efficiency, which is crucial for traceability research in blockchain. However, future research challenges involve reducing transaction length and verification time in regulated blockchain studies.

## Data Availability

The datasets generated and analysed during the current study are not publicly available due the restrictions from NUDT (National University of Defense Technology) but are available from the corresponding author on reasonable request.
